# What do pharmaceutical industry professionals in Europe believe about involving patients and the public in research and development of medicines? A qualitative interview study

**DOI:** 10.1136/bmjopen-2015-008928

**Published:** 2016-01-07

**Authors:** Suzanne Parsons, Bella Starling, Christine Mullan-Jensen, Su-Gwan Tham, Kay Warner, Kim Wever

**Affiliations:** 1 Public Programmes Team, Central Manchester University Hospitals NHS Foundation Trust and University of Manchester, Manchester Academic Health Sciences Centre, Manchester, UK; 2 Fresheyes Research, Dublin, Ireland; 3 Institute of Brain, Behaviour and Mental Health, University of Manchester, Manchester, UK; 4 Focus on the patient, Medical Platforms UK, RD Chief Medical Office, GlaxoSmithKline, London, UK; 5 Research and International Affairs, Dutch Genetic Alliance (VSOP), Soest, The Netherlands

**Keywords:** QUALITATIVE RESEARCH, SOCIAL MEDICINE, PUBLIC HEALTH

## Abstract

**Objectives:**

To explore European-based pharmaceutical industry professionals’ beliefs about patient and public involvement (PPI) in medicines research and development (R&D).

**Setting:**

Pharmaceutical companies in the UK, Poland and Spain.

**Participants:**

21 pharmaceutical industry professionals, four based in the UK, five with pan-European roles, four based in Spain and eight based in Poland.

**Method:**

Qualitative interview study (telephone and face-to-face, semistructured interviews). All interviews were audio taped, translated (where appropriate) and transcribed for analysis using the Framework approach.

**Results:**

21 pharmaceutical industry professionals participated. Key themes were: beliefs about (1) whether patients and the public should be involved in medicines R&D; (2) the barriers and facilitators to PPI in medicines R&D and (3) how the current relationships between the pharmaceutical industry, patient organisations and patients influence PPI in medicines R&D.

**Conclusions:**

Although interviewees appeared positive about PPI, many were uncertain about when, how and which patients to involve. Patients and the public's lack of knowledge and interest in medicines R&D, and the pharmaceutical industry's lack of knowledge, interest and receptivity to PPI were believed to be key challenges to increasing PPI. Interviewees also believed that relationships between the pharmaceutical industry, patient organisations, patients and the public needed to change to facilitate PPI in medicines R&D. Existing pharmaceutical industry codes of practice and negative media reporting of the pharmaceutical industry were also seen as negative influences on these relationships.

**Strengths and limitations of this study**

- One of the first studies exploring pharmaceutical industry professionals’ beliefs about patient and public involvement (PPI) in research and development of medicines across a number of European countries.
- The University of Manchester worked with research teams in all countries to ensure that a similar approach to this research was undertaken.
- This was exploratory work, and so the study sample size was relatively small. In the future, it would be interesting to explore these issues on a wider basis across the pharmaceutical industry, perhaps using a questionnaire survey.
- Despite the small sample size, we were able to identify interviewees from a number of companies and with a wide range of remits as we used a wide range of recruitment approaches.
- Owing to the nature of the topic, interviewees with experience of working with patients may have been more likely to volunteer. However, some reported little contact with patients and were not advocates of PPI which suggests that this may not have been true in all cases.

## Background

Patients have become increasingly involved in managing their own health by searching for health information and making decisions about their care along with their healthcare professionals.^1^ These developments may have changed both patients’ expectations of their healthcare professionals and of their own role in managing their health. The increased interest in and use of patient-reported outcome measures^2^ and measurement of patient experience^3^ also highlight how patients’ roles are changing from passive recipients to active participants in their healthcare.

Another indicator of patients’ changing role in their healthcare, is their increased involvement in medical and healthcare research. For example, there has been an increase in accounts of PPI in the medical research literature during the past 10–15 years.^4^ PPI in health and medical research can occur at a range of levels, from patients commenting on patient information, to being involved in study management.

In recent years, the pharmaceutical industry has become increasingly interested in how to make medicines research and development (R&D) more patient-centred. Reasons for this interest may be a response to the changing nature of patients described earlier and also due to concerns about the sustainability of medicines R&D.^5^ For example, a recent survey found that 73% of workers in the pharmaceutical industry believed that industry needs to change its relationship with patients, and 85%, that increasing the patient-centredness of medicines R&D is important for its sustainability.^6^ Concerns about the sustainability of medicines R&D have arisen due to its increasing cost relative to the number of products that reach the market, the expiration of the patents of widely used prescription medicines, and the increased regulation of the pharmaceutical sector.

Increasing PPI in medicines R&D is believed to improve the process by (1) making it more patient-centred; (2) identifying new areas of research, and promoting innovation and (3) providing new insights, identifying solutions to problems, and improving the acceptability of new medicines to patients.^7^

It is reasonable to say that PPI is currently not a well-established component of medicines R&D, although it is a growing area for which there are some well-established examples in the areas of HIV and rare diseases.^8^ There are also examples of PPI in other healthcare environments, for example, the UK's National Health Service, from which it is likely that lessons can be learnt.^9^ Historically though, pharmaceutical companies have primarily acted as financial sponsors for patient organisations, with minor involvement in the funded activity. Therefore, not surprisingly, pharmaceutical industry codes of practice discuss sponsorship rather than joint working with patient organisations.^10^ The relationship between patient organisations, patients and the pharmaceutical industry may need to change from a sponsorship to a joint working model to facilitate PPI in medicines R&D. The large number of recently developed joint working toolkits for the pharmaceutical industry and other key stakeholders in medicines R&D (academia, healthcare and patients) suggests that relationships within the environment of medicines R&D are rapidly evolving, and are of increased interest to all parties.^11^ ^12^

The European Commission and the Innovative Medicines Initiative have recognised the importance of increasing PPI in medicines R&D, and have funded the European Patients’ Academy on Therapeutic Innovation Project ((EUPATI) from 2012 to 2017 http://www.patientsacademy.eu).^13^ EUPATI aims to increase PPI in and public awareness of medicines R&D across Europe. EUPATI is a consortium project, led by the European Patient's Forum, with 30 project partners (including patient organisations, academic institutions and pharmaceutical companies). To fulfil its aims, EUPATI is developing a training course for patient experts which aims to increase their capacity to become actively involved in medicines R&D, a toolkit for patient advocates to facilitate dissemination of information on medicines R&D to the patients they represent, and an online library of medicines R&D information for the public.

This study forms part of a programme of social research conducted within EUPATI which explored key stakeholders’ perspectives on PPI in medicines R&D. As PPI in medicines R&D is currently not widely established or consistently implemented within the pharmaceutical industry, it was important to explore pharmaceutical industry professionals’ beliefs regarding increasing PPI in medicines R&D.

## Aim

The study aimed to explore European-based pharmaceutical industry professionals’ beliefs about involving patients and the public in medicines R&D.

## Methods

*Design—*One-to-one, semistructured interviews conducted as part of qualitative studies of key stakeholders in medicines R&D in three European countries.

Country-based studies were conducted in the UK, Spain and Poland. These countries were selected as they varied on:

1. Date of entry into the European Union
2. Government health expenditure
3. Employment rate

EUPATI is also being established at a local level in these countries, where it will implement and disseminate its materials.^14^

The UK study was conducted by the University of Manchester, which commissioned GFK (Gesellschaft für Konsumforschung) Ad Hoc research,^15^ and CEM Market and Public Opinion Research^16^ to conduct the fieldwork in Spain and Poland, respectively. GFK and CEM received extensive study briefings and had regular contact with the University of Manchester team during the fieldwork to ensure that similar approaches were used in all countries.

Within each country, the perspectives of patient representatives, patients and the public, clinical research professionals, healthcare policymakers, and pharmaceutical industry professionals were explored. However, this paper just reports the data from professionals in the pharmaceutical industry.

## Recruitment and sampling

Within our ethics committee applications for this work, we specified that either focus groups or one-to-one interviews could be the data collection approach used, depending on participant preference. Initially, we planned to hold focus groups with pharmaceutical industry professionals at two EUPATI-organised conferences. However, despite several attempts, we were unable to recruit to these groups. We found that a one-to-one interview approach (face-to-face or telephone) was considered more appropriate by interviewees, and we hypothesised that this may be for reasons of convenience and confidentiality.

We recruited interviewees by advertising the study internally within pharmaceutical companies, on EUPATI's LinkedIn and Facebook pages and via Farmaindustria and the European Federation of Pharmaceutical Industries and Associations. Some interviewees were also identified via snowballing. We used a purposive sampling approach using interviewees’ professional role and size and type of company as our sampling criteria. An interview topic guide was developed which explored the following:

- Beliefs about patients’ and the public's knowledge and understanding of medicines R&D
- Beliefs about patients’ and the public's information needs regarding medicines R&D
- Experience of PPI in medicines R&D, including beliefs about PPI
- Beliefs about barriers and facilitators to developing patient information about medicines R&D and to PPI.

The interview topic guide is included as online supplementary appendix 1 of this paper.

The topic guide was developed by reference to a literature review on this area conducted by the needs assessment work package team, and also via brainstorming and discussion within this team and the wider project consortium.

Interviewees were asked to talk about their own views on this issue rather than about their company's position. They were also asked to talk about their own experiences and perceptions of PPI within their companies.

## Transcription, translation and data analysis

Interview recordings were transcribed verbatim. Spanish and Polish recordings were translated into English. All transcripts were pseudo-anonymised, that is, pseudonyms of names and organisations were created. Thematic data analysis was carried out using the Framework approach.^17^ We decided to use the Framework approach, as this was a piece of applied qualitative research, and using Framework allowed both a priori and emergent concepts to be included in the analysis. Therefore, using this approach enabled us to explore issues that were of particular importance to the wider EUPATI project, and incorporate them into the analysis as well as incorporating emergent concepts from the data. The transparent and practical nature of the Framework approach also helped to facilitate the involvement of the large needs assessment work package team in the data analysis, and it enabled us to compare the range of perspectives within each stakeholder group, and the perspectives of different stakeholders on the same themes within the country-based studies.

Qualitative data analysis was led by the University of Manchester (SP, S-GT and BS), and primarily by SP who has 15 years’ experience in qualitative research. However, all members of the needs assessment work package were involved in the data analysis. For example, all were involved in developing the initial topic guide; SP, BS, KW and CM-J were involved in discussing the fieldwork approach and interview context and findings with the research teams in the UK, Poland and Spain.

The initial thematic framework was developed by SP, BS, CM-J, KW and S-GT, and this was then discussed and refined within the wider needs assessment group. SP coded the transcripts with around 50% being reviewed by S-GT. Coded transcripts and analysis drafts were discussed within the wider needs assessment group.

## Ethical issues

Ethical committee approval for the study was obtained from the Hampstead NHS Research Ethics Committee and the University of Manchester Research Ethics Committee. Informed consent was obtained prior to all interviews.

## Results

Twenty-one pharmaceutical industry representatives participated (four from the UK, five with a pan-European remit, four from Spain and eight from Poland) (table 1).

**Table 1:** Characteristics of interviewees

	Gender	Job function	Country
Interviewee 1	Female	Market research	UK
Interviewee 2	Male	Regulatory affairs	UK
Interviewee 3	Male	Public affairs	UK
Interviewee 4	Female	Communications	UK
Interviewee 5	Female	Medical affairs	Pan-European
Interviewee 6	Male	Patient relations	Pan-European
Interviewee 7	Male	Policy and patient advocacy	Pan-European
Interviewee 8	Female	Government affairs and lobbying	Pan-European
Interviewee 9	Female	Communications	Pan-European
Interviewee 10	Male	Clinical development	Spain
Interviewee 11	Female	Clinical research	Spain
Interviewee 12	Female	Clinical development	Spain
Interviewee 13	Female	Patient advocacy	Spain
Interviewee 14	Male	Public relations	Poland
Interviewee 15	Male	Medical affairs	Poland
Interviewee 16	Female	Head of clinical studies	Poland
Interviewee 17	Male	Clinical trials director	Poland
Interviewee 18	Male	Registration of services	Poland
Interviewee 19	Male	Clinical development	Poland
Interviewee 20	Female	Clinical research associate	Poland
Interviewee 21	Female	External affairs and public relations	Poland

The following themes were identified.

Beliefs about

- Whether patients and the public should become involved in medicines R&D
- Challenges to and facilitators of PPI in medicines R&D
- How the relationship between the pharmaceutical industry and patient organisations influences PPI in medicines R&D

Illustrative quotes for the key themes are presented in table 2.

**Table 2:** Key themes and illustrative quotes

Themes	Findings	Illustrative quotes
Beliefs about PPI in medicines R&D	Views varied widely Positive and some ideas about how to implement Positive and little idea about how to implement Negative—believe that there is little role for the public in medicines R&D	*“But you could also bring a couple of patients into that (physician advisory board) who could say, well that's all well and good from a physician's perspective, but actually the way that would be as an experience for the patient, I'd recommend you did this”.* **Interviewee 2** *“They could influence the manner of conducting studies as well as planning the studies”* **Interviewee 5** *“In general, such role would be slight; because the place where patients can play a role are the studies, so maybe some sort of cooperation regarding the studies, patients’ associations might be involved in it”.* **Interviewee 15**
*Challenges and facilitators to patient involvement—Patient related issues*		
Patient and public knowledge and awareness of medicines R&D	Beliefs about patients and the public's information needs Believe a detailed level of knowledge is needed Believe that a detailed knowledge is medicines R&D is not required	*“There are lots of things that they understand but they don't always appreciate, I think the constraints in which we work. For example, when we have commitments for data we need to bring them in a certain timeframe, because we also have for internal rules and budgeting rules”.* **Interviewee 5** “*If it's to inform the study and the design then you probably need a fairly detailed level of knowledge about how the disease works, how you might interpret it and practical side of things. So I would have thought that its fairly feasible but it does take the right individual and you have to clarify what you are looking for from them”.* **Interviewee 2** *“I think that the public should know as much as it is interested in knowing. The patients don't really need to know about the process of developing a medication. In this knowledge, the most important aspect is the final document which evaluates the benefits and the risks for the patients and the manner in which we arrive at these benefits and risks”*. **Interviewee 18**
Interest in, and engagement with, medicines R&D	Believe that patients knowledge and interest in medicines R&D varies depending on their individual characteristics, and that in many quarters that knowledge and awareness is growing Some felt that patients and the public don't think about medicines R&D until they need to Even if high-quality information is available many people won't attend to it One explanation for lack of engagement might be negative public perceptions of industry which many interviewees felt were shaped by what they felt was an ill-informed mass media	*“I think that everything depends on the group of patients. Definitely, there's greater awareness among young patients. Especially with respect to chronic diseases, which do not have treatment that would be 100% effective? Currently, our company is also dealing with rare diseases where the parents’ awareness is much greater. Young people have contact with the world; they have access to the Internet, they use it and they search for information”.* **Interviewee 17** *“That is very difficult to say. I do not know if our society, in general, feels a need to have that information, they do not really care”.* **Interviewee 10** *“It seems to me that the society is completely deprived of immunity to negative sensational stories of this type. This may partly be the result of limited knowledge”*. **Interviewee 2**
*Pharmaceutical industry-related issues*		
Understanding of patient involvement	Interviewees felt that the aims, functions and values of patient involvement were poorly understood within the pharmaceutical industry	*“And I'm certainly an advocate for them being more involved at the regulator side, but also why not work more closely with industry so industry knows before the regulators tell them the patient perspective on whether they come to market is X, and we've got a closer voice for that”.***Interviewee 2**
Operationalising patient involvement	Challenge of working with different-sized patient organisations with different agendas Challenges of conflicting agendas and agenda-focused contribution of patients Beliefs about how and when patients should become involved	*“If you go to industry, you got to industry, and they're either a big company or they're a small company, but when you go to the charities they all have different aims and objectives, they all have different requirements and different specialisms, they all have different levels of understanding of what's going on”.* **Interviewee 4** “*It's nearly a conflict of interest, really with patient based organisations, that's what they want is to have some control and to be able to drive research in the direction they think it's most important and should be going. I think it's especially difficult in the post-marketing space where as industry we have to generate evidence for EMA or payers. The patient organisations need to understand these commitments so that they can integrate these commitments into their plans. I think sometimes that what is not always well understood is the environment in which pharmaceutical industries have to operate”.* **Interviewee 5** *“It requires from the patients to be able to take a perspective that is not necessarily his own perspective but more being a representative of a group of patients affected by the same disease”*. **Interviewee 5** *“I personally think that if we're able to make drug companies a little less mean and leave and were able to spend some time so that there was that kind of explanation given to the patient that would be great.”* **Interviewee 6**
*Relationship between the pharmaceutical industry and patient organisations*		
Beliefs about current relationship with patient organisations	Belief that their current contact with patients was relatively industry led—sponsorship or asking for specific information from patients Beliefs about the changing relationship between patients and industry	*“I'd say it was still a journey because some of the patient groups. The old model was industry gave them money and the bigger the company the more money you got and it didn't really matter what it was for. And now we are looking specifically at funding projects.”* **Interviewee** **4** *“We work with patient associations and the last one that I did started in 2001, and it's amazing how things have changed in the last 12 years, in that the work that we were able to do together with the patient association then, would be completely not allowed with today's world. It was a much more intimate involvement in what we were doing and now whenever we discuss what we're doing; it's considered that we might be enticing them to take a drug and all this kind of study.”***Interviewee 6**
Influences on current relationship	Belief that patients and the public's negative perceptions of the pharmaceutical industry are strongly shaped by negative reporting of the pharmaceutical industry Little available medicines R&D information and that which is in negative and from the media Financial drivers	*“So you hear negative perceptions and stories about trying to get drugs sold in various countries. The big fine, X has had in the country, so you hear about that and then you hear that drugs are high priced and Bad Pharma, but you don't really hear good news and drug development in its simplest form where you produce something is not really praised in the same way.”* **Interviewee 3** *“In reality, the journalists are not really interested in the problem as such; they are only searching for a sensation”.* **Interviewee 17** *“Doctors know that it's safe and a patient may resign at any moment. It's not like you sign a document and you have to participate in it until the end. But everything boils down to education. The knowledge is based on myths and stereotypes and it is very limited.”* **Interviewee 14** *“I've been working with the patient groups, the ones that I currently have a relationship with and with new ones that approach us and they sort of say, well, that's really unusual because normally pharma just kind of gives us money. And I say I can't give you guidance as to whether or not it'll be accepted; it all depends on is it compliant and is it appropriate, as deemed by our independent committee. It's been interesting, because some of the patient groups have sort of said, but, that sounds awfully complicated, and others, the larger ones, say actually, that makes perfect sense.”* **Interviewee 4**

R&D, research and development.

### Beliefs about whether patients and the public should become involved in medicines R&D

Interviewees’ beliefs about PPI in medicines R&D could be split into three types: (1) those that were positive with ideas about PPI, (2) those who were positive but with no ideas about PPI and (3) those who believed patients should not become involved in medicines R&D.

Interviewees in Spain and Poland expressed more uncertainty about the benefits and value of PPI than those in the UK and with pan-European roles. Unsurprisingly, those who were positive were also more likely to have ideas about how to involve patients and the public, for example, adapting existing approaches by which other stakeholders are involved. However, the majority of interviewees believed that there were currently few plans within the pharmaceutical industry regarding PPI. In terms of job role, those who had ideas about PPI predominately worked in patient advocacy, market research and policy, that is, roles where they had patient contact or where PPI had been discussed as a policy issue. Those who expressed uncertainty predominately worked in medical affairs and clinical development. Interviewees still felt positively about PPI in medicines R&D even if they currently had few ideas about how to implement it. Therefore, job function was not always related to beliefs about PPI in medicines R&D.

### Challenges to, and facilitators of, PPI in medicines R&D

**Figure 1:**
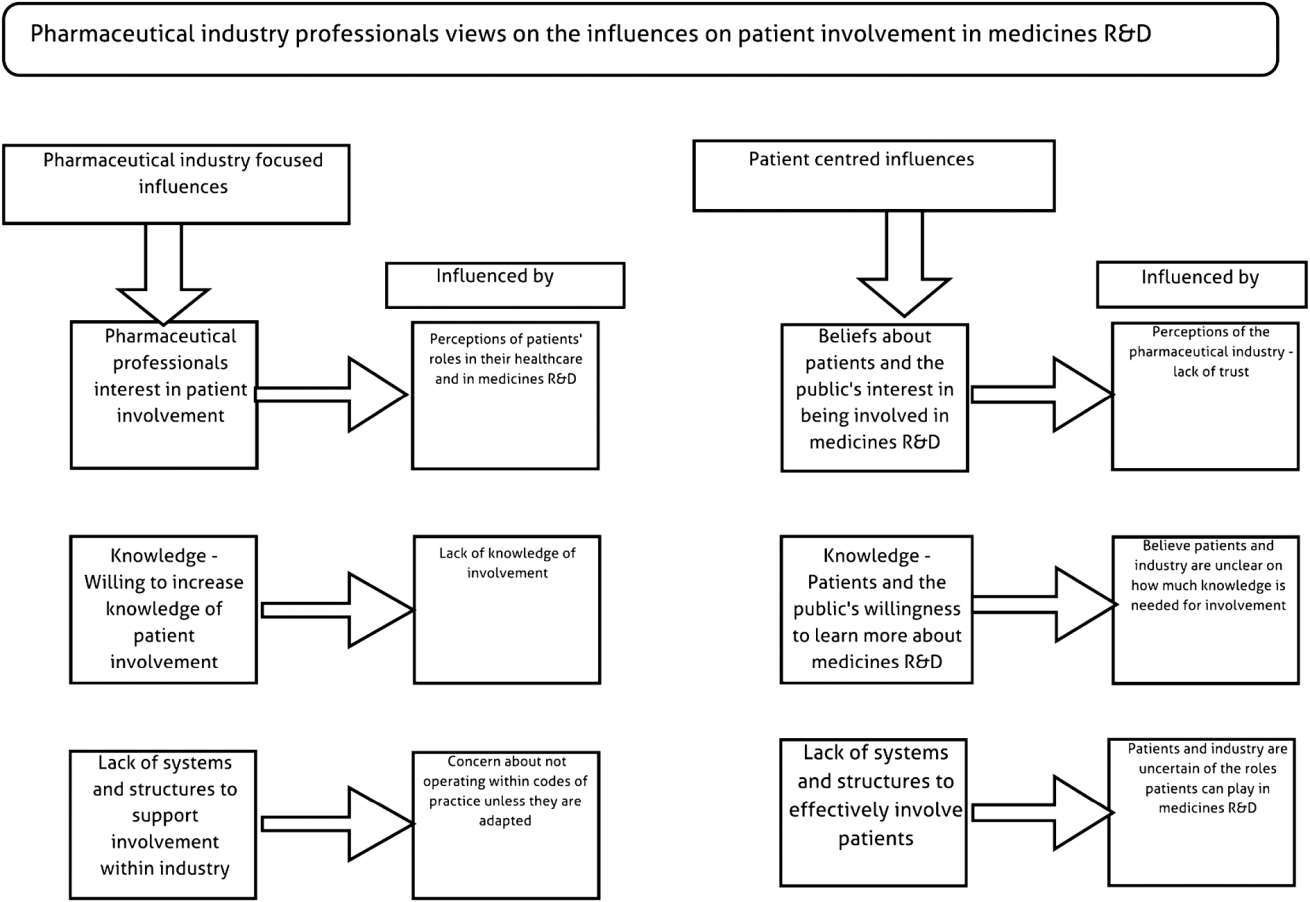
Pharmaceutical industry professionals' views on the influences on patient involvement in medicines research and development. R&D, research and development.

Interviewees described a number of challenges to, and facilitators of, PPI which can be organised into patient-related and pharmaceutical industry-related issues. These challenges and facilitators are also described in figure 1.

## Patient-related issues

### Knowledge of medicines R&D

Interviewees in all job roles believed that patient organisations without experience of working with the pharmaceutical industry were more likely, to have little knowledge of medicines R&D, and to hold negative views of the pharmaceutical industry. They felt that both these factors could deter patients from becoming involved in medicines R&D. Those who worked within patient advocacy and market research believed that patients’ views were likely to change if they gained experience of working with the pharmaceutical industry, in some cases they were speaking from experience of this. However, interviewees believed that, without such experience, patients’ knowledge of medicines R&D would remain low and continue to be shaped by what they felt was exposure to negative media coverage of the pharmaceutical industry. All interviewees acknowledged though that some patient organisations already had good knowledge of medicines R&D and experience of working with the pharmaceutical industry, for example, those representing patients with HIV or rare conditions.

Therefore, to facilitate PPI and increase patients’ knowledge of medicines R&D, all interviewees, except those who did not believe that patients should be involved, believed that high-quality information in medicines R&D was needed. Those who felt positively about PPI also believed it was important for the pharmaceutical industry to identify at what points patients could become involved in medicines R&D, and what roles they could play to help inform patients’ expectations of their involvement.

However, interviewees’ beliefs about the level of knowledge required for PPI varied. Some, even if they were positive about PPI, believed that only patients with medical knowledge should be involved, whereas others believed that just a basic knowledge of medicines R&D was necessary for PPI, as patients have their own knowledge and experience to contribute. This lack of agreement may be indicative of the apparent uncertainty regarding PPI expressed by the study interviewees.

## Patient-related issues

### Interest in medicines R&D

Unsurprisingly, all interviewees believed that patient organisations’ level of interest in medicines R&D influenced their decision to become involved. They believed that patient organisations were less likely to be interested if there was a well-established treatment for the condition, whereas those who were struggling with access to innovative treatments were likely to be very interested. Regardless of their beliefs about PPI in medicines R&D, interviewees believed that the public were unlikely to be interested in medicines R&D unless they or a family member became unwell, and that this lack of interest could make it challenging to increase PPI and public awareness of medicines R&D.

Those who felt positively about PPI and who wanted to increase the amount of PPI in medicines R&D, also attributed patients’ and the publics’ lack of interest in medicines R&D, to distrust in the pharmaceutical industry, and to a lack of good quality, unbiased and interesting information about this. Therefore, they believed that the provision of good quality information, and exploring ways of increasing patients’ and the public's trust in the pharmaceutical industry were essential to raising interest in medicines R&D.

Again, those who wanted to increase PPI in medicines R&D, and particularly those working in policy and public affairs, believed that it might be beneficial for the pharmaceutical industry to work with the media to improve their understanding of, and communication of, news about medicines R&D, and that this, in turn, might help to increase public trust. They suggested this as they believed that exposure to negative media stories about the pharmaceutical industry was a key source of information for, and an influence on, the views of patients and the general public. There may be issues with the media's receptivity to such suggestions though.

## Pharmaceutical industry-related issues

### Understanding of, and interest in, patient involvement

As described earlier, all interviewees described great uncertainty within the pharmaceutical industry regarding the aims, function and value of PPI in medicines R&D. Therefore, those who felt positively about PPI believed that there was a need to increase knowledge and understanding of PPI within the pharmaceutical industry, to improve its readiness and ability to involve patients. There was some uncertainty though regarding who should take responsibility for this within a company, or within trade organisations, and also regarding the structures, systems and resources that are needed to support PPI.

Those who were less positive did not perceive a need to increase the pharmaceutical industry's knowledge and understanding of PPI.

However, as described earlier, some interviewees believed that patients’ primary role in medicines R&D should be that of a research participant, and they did not see a role for patients in being more actively involved. Where such attitudes exist, it may be challenging to implement PPI. In such situations, cultural changes regarding how patients’ roles in their healthcare are viewed may be needed. However, such changes may take time and be influenced by a wide range of factors not all of which are specific to the pharmaceutical industry.

## Pharmaceutical industry-related issues

### Beliefs about implementing PPI in medicines R&D

As described earlier, regardless of their feelings about PPI, interviewees believed there was little agreement and understanding within the pharmaceutical industry on how to implement and organise PPI. For example, they believed there was little clarity regarding which stages patients could be involved in, which types of patients should be involved, and on how to support PPI in medicines R&D.

Those who felt positively about PPI in medicines R&D and who were interested in working out how to actively involve patients and the public, also believed that there was little agreement about which types of patient organisations should be involved in terms of their size and characteristics, for example, some believed that it would be easier for the pharmaceutical industry to work with just large organisations, whereas others felt that the size of the patient organisation involved should depend on what was being asked of them.

### How the nature of the relationship between the pharmaceutical industry and patient organisations influences PPI in medicines R&D

One explanation for interviewees’ lack of awareness and experience of PPI may have been a lack of patient contact during their working lives. As one might expect, the extent to which interviewees had contact with patients and patient organisations varied greatly with those working in market research and patient advocacy reporting the greatest contact and those working in regulation the least. However, a lack of experience of working with patients was not always related to negative views about PPI. A number of interviewees with little experience were very positive about learning more about PPI and involving patients and the public in their work.

Among those with experience of working with patients, two approaches were described. First, where patient organisations were asked to input into specific aspects of pharmaceutical industry-led projects, and second, where interviewees were asked to provide financial sponsorship to patient organisation-led initiatives. No examples of joint working with patients were described by the interviewees. That is not to say that they do not exist, though.

Interviewees felt that they needed to increase and improve their contact with patients to facilitate PPI in medicines R&D. They believed that it would be difficult for patient organisations and pharmaceutical companies to work together effectively without developing a closer relationship. For example, interviewees suggested that developing increased clarity and agreement with patient organisations regarding their role in medicines R&D was essential to facilitating better relationships and increasing trust.

Despite believing that change was necessary, interviewees also felt that it might be challenging to implement due to the current lack of agreement and awareness within the pharmaceutical industry regarding PPI in medicines R&D

## Discussion

### Beliefs about PPI in medicines R&D

In the main, interviewees were positive about increasing PPI in medicines R&D, although varied in their confidence in, and experience of, involving patients. Thus, it may be important to identify case studies of good practice in PPI in medicines R&D, and communicate them widely throughout the pharmaceutical industry and to patient organisations. This may help to make PPI a more tangible concept to these groups, and to promote awareness of the value and benefits of PPI in medicines R&D. Several case studies of PPI in medicines R&D were identified as part of EUPATI. However, this was challenging due to little information being available about many.

When identifying examples of good practice in PPI, case studies will need to be evaluated to identify whether and how they constitute good practice. However, identifying good practice in PPI in healthcare research can be challenging in general, not just in the pharmaceutical industry.^18^ Therefore, it may be important to develop good practice standards for PPI in medicines R&D.

Some interviewees also spoke about their need for training and support on how to effectively involve patients and the public. This has also been identified as a need for PPI in other healthcare areas.^19^

A minority of interviewees appeared to have little awareness of, or could not see the value of PPI in medicines R&D. One explanation for this may be that in their countries, there may not have been a strong culture of consulting the public about their healthcare. For example, the doctor–patient relationship has been shown to vary across Europe, with patients in some countries being more likely to be viewed as recipients of, rather than active participants in, their care.^20^ If patients are viewed primarily as care recipients then this may decrease both patients and pharmaceutical industry professionals’ expectations that patients can, and should be involved in medicines R&D.

Where such views persist, considerable culture change regarding how patients’ roles in their healthcare are viewed may be necessary before PPI can be widely implemented. However, such change will be gradual, take time, and be influenced by a range of factors, not all related to the pharmaceutical industry. It will be important to identify powerful change agents and champions of PPI working both within and outside the pharmaceutical industry to drive change and influence opinion in these situations.^21^

### Challenges to and facilitators of PPI in medicines R&D

Interviewees believed that negative public perceptions of the pharmaceutical industry were a key challenge to PPI in medicines R&D. Some attributed this to patients and the public's lack of knowledge and experience of medicines R&D, and to their belief that their knowledge was often informed by an ill-formed mass-media. Therefore, as EUPATI intends, increasing the amount and availability of high-quality information on medicines R&D may increase patients’ and the public's knowledge of this area. However, to ensure that information is used, it will be essential that it is widely communicated, presented in an interesting fashion and supported by trusted healthcare information providers for patients and the public to use.

As many interviewees felt that negative media reports about the pharmaceutical industry were a major source of public information about this area, then the provision of good quality, impartial information may also help to increase public trust. However, it may be useful to examine the extent to which media reports on medicines R&D are actually negative and influence public views. It may also be interesting to explore the media's receptivity to working with the pharmaceutical industry to improve their knowledge of medicines R&D.

In the future, it may also be important to explore whether patients’ and the public's perceptions of the pharmaceutical industry are as negative as our interviewees believed. Indeed, there is evidence to suggest that the public's views may be more appropriately classified as mixed, rather than negative. A 2013 survey found that the public greatly valued the pharmaceutical industry's contribution to the economy and to improving healthcare in general.^22^

It is important to note that increasing trust in the global pharmaceutical industry is likely to take considerable time, and be influenced by many factors other than the provision of information. Nonetheless, projects like EUPATI may act as a catalyst for change. For example, EUPATI has already convened a meeting of its pharmaceutical industry partners to discuss many of the issues identified in this research.^23^

Another barrier to PPI from the perspectives of those who were positive about it was the lack of readiness and receptivity within the pharmaceutical industry to involve patients and the public in their work. Therefore, it may also be important for pharmaceutical industry trade organisations and others to develop and provide guidance on this area. Increasing PPI in medicines R&D is also likely to require both financial and personnel resources and the development of systems and structures to support involvement. Lessons may be learnt regarding this from elsewhere in healthcare. An important recent development in tackling industry's receptivity to PPI is the Patient Focused Medicines Development Initiative which may be important in increasing patient involvement in medicines R&D. This is a US–European partnership of patients and senior representatives of multinational pharmaceutical companies and has the objective of developing and sharing a master framework for systematic and integrated patient involvement in medicines R&D.^24^

### Relationship between the pharmaceutical industry and patient organisations

Those who were interested in increasing PPI in medicines R&D believed that good relationships between pharmaceutical companies and patient organisations were essential to PPI. However, many felt that developing good relationships was difficult as they had little contact with patients and the public in their day-to-day working lives. Some attributed this lack of contact to their need to work within existing codes of practice. Therefore, our findings suggest that revisions to the codes may be useful, for example, to provide guidance on the conduct of the pharmaceutical industry when involving patients and the public,^10^ and to facilitate PPI if pharmaceutical industry professionals are comfortable that they are acting within existing codes of practice. It may also be beneficial to involve patients in revising the existing codes of practice to ensure that any revisions are acceptable to patients.

This research was undertaken within the context of a public–private partnership initiative with the aim of increasing PPI in medicines R&D by the provision of information and training. Therefore, the multistakeholder consortium was interested in finding out the challenges to developing information and to facilitating PPI in medicines R&D. The group involved in undertaking this work represented all key stakeholder groups within the public–private partnership (academia, patient organisations and pharmaceutical industry), and so all relevant perspectives could be incorporated into the data analysis. Work was led by the University of Manchester, and by SP and BS who had previously not undertaken any Pharmaceutical Industry-based research. Therefore, it was possible to approach the research with relatively few preconceptions about the area. SP had some initial awareness of the poor public image of the pharmaceutical industry, but her knowledge of the pharmaceutical industry was low. GFK and CEM had both undertaken work for pharmaceutical companies before, for example, work around reputation, but they had not explored PPI in medicines R&D, and so this area was new to them. Professionals in the pharmaceutical industry were asked to give their personal rather than professional views, on a one-to-one basis, to an individual who worked outside the pharmaceutical industry which we believed helped to increase their openness, and also willingness to express scepticism about this area.

## Conclusions

PPI in medicines R&D is an emerging area which is likely to be challenging to implement in the global pharmaceutical industry due to different working practices, regulatory environments and interpretations of codes of practice. Varying views regarding patients’ roles in their healthcare across different countries may also present a challenge. Increasing PPI in medicines R&D is likely to take some time and resources, particularly in the development of patient information, as well as potential amendments to existing pharmaceutical industry codes of conduct.

However, initiatives such as the EUPATI project may act as a catalyst for PPI in medicines R&D via the provision of information for patients and the public. Other catalysts may be revisions of existing pharmaceutical industry codes of practice and communication of these revisions. Working with the media to increase their understanding of the pharmaceutical industry may also be important, but challenging and time consuming.

Finally, it may be important to identify and widely communicate examples of good practice in PPI within the pharmaceutical industry as, currently, to many of those interviewed, PPI felt like a good but intangible idea.
